# Early stage follicular lymphoma: what is the clinical impact of the first-line treatment strategy?

**DOI:** 10.1186/1756-8722-6-45

**Published:** 2013-07-01

**Authors:** Anne-Sophie AS Michallet, Laure L Lebras, Deborah D Bauwens, Fadhela F Bouafia-Sauvy, Françoise F Berger, Christelle C Tychyj-Pinel, Anne A D’Hombres, Gilles G Salles, Bertrand B Coiffier

**Affiliations:** 1 Department of Hematology, Hospices Civils de Lyon, Centre Hospitalier Lyon Sud, Pierre Bénite, France; 2 Université de Lyon, Faculté de Médecine Lyon-Sud Charles Mérieux, Pierre Bénite, France; 3 Clinique Universitaire Saint Luc, UCL, Woluwe Saint Laurent, Belgium; 4 Department of Anatomopathology, Hospices Civils de Lyon, Centre Hospitalier Lyon Sud, Pierre Bénite, France; 5 Department of Radiology and Nuclear Medicine, Hospices Civils de Lyon, Centre Hospitalier Lyon Sud, Pierre Bénite, France; 6 Department of Radiotherapy, Hospices Civils de Lyon, Centre Hospitalier Lyon Sud, Pierre Bénite, France; 7 Service d’Hématologie Clinique, Centre Hospitalier Lyon Sud, 165 chemin du Grand Revoyet, F-69310 Pierre Bénite, France

**Keywords:** Follicular lymphoma, Chemotherapy, Complete response, Rituximab, Radiotherapy

## Abstract

**Background:**

Less than 20% of patients with follicular lymphoma (FL) present with Ann Arbor Stage I or II disease at diagnosis. Numerous therapeutic options exist, however radiation therapy is considered the standard of care for early-stage disease based on single-institution or retrospective series. Our aim was to revisit the outcome of patients with localized FL in the rituximab era.

**Patients and Methods:**

We analyzed the characteristics and outcomes of 145 early-stage FL patients, who were retrospectively divided into six groups according to their initial treatment: watchful waiting (WW), chemotherapy alone (CT), radiotherapy alone (RT), combined radiotherapy and chemotherapy (RT-CT), rituximab alone (Ri), and immunochemotherapy (Ri-CT).

**Results:**

Of the 145 patients, 84 (57.9%) had stage I disease and 61 (42.1%) stage II. The complete response (CR) rate varied from 57% for the Ri group to 95% for the RT-CT group. Overall survival (OS) at 7.5 y of patients treated after 2000 was better than that of those treated prior to 2000. OS did not significantly differ from one treatment to another. In contrast, a significant difference was found for progression-free survival (PFS) at 7.5 y, which favored Ri-CT (60%) therapy versus the others (*p=*0.00135).

**Conclusion:**

Delayed therapy initiation was associated with a similar OS than that observed in patients receiving immediate intervention. The “watchful waiting” strategy may thus be proposed as first-line therapy, similar to stage III and IV FL patients with a low tumor burden. However, when treatment is required, immunochemotherapy appears to be the best option.

## Background

Follicular lymphoma (FL) is the most frequently encountered type of indolent lymphomas. Indolent lymphomas are considered to be an incurable malignancy with a relapsing and remitting course. For the vast majority of patients, the disease is managed over many years [1]. Most patients are diagnosed with FL at an advanced stage, with <20% presenting with either stage I or II. In the small proportion of patients (15-20%) diagnosed with localized Ann Arbor stage I or II disease, the treatment of choice remains poorly defined. A wide range of management options exists, from extended or involved field radiotherapy, which remained the mainstay treatment [2-4], to the use of combined radiochemotherapy [5,6] or the “watchful waiting” approach [7,8]. As there has been no randomized study, it is difficult to claim that one strategy is better than another [9]. An Italian expert panel published guidelines for the management of nodal indolent lymphomas [10]. They recommended involved field (IF) radiotherapy in low tumor burden localized disease, with adjuvant chemotherapy being reserved for cases of high tumor burden or high-risk disease (FLIPI score >2). The experts did not retain the eventuality of a “watchful waiting” option in a low burden case, which was associated with a good outcome in the Stanford experience [8]. More recently, a report mentioned that upfront RT was associated with improved OS compared with alternate management approaches (surveillance, epidemiology and end results database analysis) [11]. At present, new low-toxicity therapies that prolong progression-free survival periods and the interval period to new anti-lymphoma therapies are available and include rituximab monotherapy or the combination of rituximab plus chemotherapy [12]. However, the impact of current therapies on early Stage FL is still unclear. The important question concerning the curability of this disease using a radical treatment remains unanswered.

We report the results of a retrospective study, including 145 patients diagnosed with localized-stage FL (stage I and II). These patients were treated with one of the six following options as first-line therapy in compliance with the physician’s decision: watchful waiting (WW), chemotherapy alone (CT), radiotherapy alone (RT), a combination of radiotherapy and chemotherapy (RT-CT), immunotherapy alone (Ri), and immunochemotherapy (Ri-CT). With a median follow-up period of 7 years, we determined the clinical characteristics of each group and analyzed the overall and progression-free survival (OS and PFS) in order to determine the best approach.

## Results and discussion

Of the 145 patients, 84 (57.9%) had Stage I disease and 61 (42.1%) Stage II. Only 22 (15.2%) presented bulky disease. Twelve patients were classified as having Stage IE or IIE. FLIPI scoring showed that 93% of the patients were in the low-risk group with a score of 0–2 (FLIPI score was 0–1 in 116 pts and 2 in 29 pts) and no patient had a score of >2.

**Figure 1:**
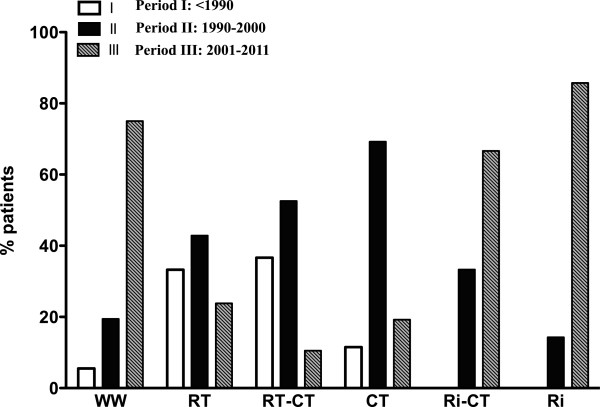
**Repartition of treatment over time (% of patients).** Three time intervals: Period I: <1990, Period II: 1991–2000, and Period III: 2001–2011.

Patient management significantly varied over the different time periods (Figure 1): Period I: <1990; Period II: 1991–2000, and Period III: 2001–2011. RT use decreased (43% for Period I versus 24% for Period III), whereas delayed therapy increased from 19.5% for Period I to 75% for Period III. The use of Ri and Ri-CT increased rapidly over Period III.

### Watchful waiting strategy (WW)

Thirty-six patients (25 females and 11 males) did not receive any treatment at the time of diagnosis. The median age was 58 years, with 12 patients (33.3%) aged over 65 years. Twenty-two patients (61.1%) had stage I disease and 14 (38.9%) stage II disease, while no patients had bulky disease. Only two of these patients exhibited abnormal LDH levels.

### Radiation therapy alone (RT)

Twenty-one patients (9 females and 12 males) were treated with RT alone. The median age was 51 years, with only four patients (19%) aged over 65 years. Seventeen patients (90.5%) had stage I disease and only two (9.5%) had stage II disease. None of them had bulky disease. Thirteen patients (65%) had a FLIPI score of 0, and five (30%) had a score of 1. No patient exhibited elevated β2-microglobulin levels or abnormal LDH levels. Five patients received extended RT and 16 received IF radiotherapy. Radiation doses ranged from 40 to 50Gy.

### Chemotherapy alone (CT)

Twenty-six patients (12 females and 14 males) were treated with CT alone. The median age was 52.5 years, with eight patients (31%) aged over 60 years. Nine patients (34.6%) had stage I disease and 17 (65.4%) stage II, and the majority (17 patients) had no bulky disease. Twenty patients (77%) had normal LDH levels. In terms of the chemotherapy regimen, all patients received an anthracycline-containing regimen (CHOP or CHOP-like regimen).

### Immunotherapy (Ri)

Only seven patients (4 females and 3 males) were treated with Ri alone as the single agent. Four injections were administered weekly with no further maintenance. The median age was 55 years, with 28.6% being older than 65. Three patients had stage I disease and four stage II. The majority of the patients had no bulky disease (71.4%).

### Combination of chemotherapy and radiotherapy (CT-RT)

Eighteen patients (10 females and 9 males) were treated with various CT regimens (CHOP and CHOP-like regimens) plus RT. The median age was 51 years; none of these patients were older than 60 years. Seventeen (89.5%) had stage I disease and only 2 (10.5%) had stage II. The majority of patients (95%) had no bulky disease with a FLIPI score of 0. Fourteen patients had normal LDH levels. Three patients received additional extended RT, and 15 patients were given IF radiotherapy.

### Immunochemotherapy (Ri-CT)

Thirty-six (17 males and 19 females) were treated with the combination of both Ri and CT, principally with the CHOP regimen. The median age was 56 years with only four patients being older than 65. Fifteen (41.7%) patients had bulky disease, and 17% had an abnormal LDH level at the time of diagnosis. Twenty-two patients had stage II (61%) disease and 14 stage I (39%).

### Comparison of the six groups

Median age, sex ratio, number of patients over 60 years, stage of disease, and LDH levels were analyzed for the six groups (Table 1). There was a significant difference in terms of disease stage when comparing the RT based group (RT and RT+CT) group against the Ri based group (Ri-CT and Ri) with a large majority (90%) of Stage I disease in the RT-based groups (*p=0,018*) without any bulky disease (*p= 0,05*). Finally, the Ri-CT group had a higher number of adverse features: 41.7% of bulky disease and 16.7% of abnormal LDH levels.

**Table 1:** Clinical characteristics of the population according to type of treatment

	**W and W**	**CT**	**Ri-CT**	**RT-CT**	**RT**	**Ri**
Number	36	26	36	19	21	7
Median age	58	52.5	56	51	51	55
>65 years	12 (33.3%)	8 (30.8%)	4 (11.1%)	0 (0%)	4 (19%)	2 (28.6%)
Gender						
M	11 (30.6%)	14 (53.8%)	17 (47.2%)	9 (47.4%)	12 (57.1%)	3 (42.9%)
F	25 (69.4%)	12 (46.2%)	19 (52.8%)	10 (52.6%)	9 (42.9%)	4 (57.1%)
Stage						
I	22 (61.1%)	9 (34.6%)	14 (38.9%)	17 (89.5%)	19 (90.5%)	3 (42.9%)
II	14 (38.9%)	17 (65.4%)	22 (61.1%)	2 (10.5%)	2 (9.5%)	4 (57.1%)
Bulky						
No	36 (100%)	17 (65.4%)	20 (55.5%)	18 (94.7%)	21 (100%)	5 (71.4%)
Yes	0	4 (15.4%)	15 (41.7%)	1 (5.3%)	0 (0%)	2 (28.6%)
LDH						
N	N	31 (86.1%)	27 (75%)	14 (73.7%)	11 (52.4%)	7 (100%)
> N	> N	1 (3.9%)	6 (16.7%)	2 (10.5%)	0 (0%)	0 (0%)

*WW* watchful waiting, *CT* chemotherapy, *RT-CT* radiotherapy and chemotherapy.

Ri: immunotherapy, and Ri + CT: immune-chemotherapy.

### Response rates and disease progression

The CR rate varied from 57.1% for Ri to 95% for the RT-CT group, with 69% for CT, 75% for Ri-CT, and 81% for RT alone, as shown in Table 2. As expected, the proportion of the PR was higher in the Ri group (43%). With a median follow-up interval of 7 years, the relapse rate was significantly lower in the Ri-CT group (40%) versus the RT-based group (90.5% for RT, 84% for RT-CT; *p= 0,048*); and 69% for CT as shown in Table 2.

**Table 2:** Response to treatment, disease progression, and survival

	**W and W**	**CT**	**Ri-CT**	**RT-CT**	**RT**	**Ri**
**Responses**						
**CR**		69.2%	75%	94.7%	80.9%	57.1%
**PR**		19.2%	16.7%	5.3%	9.5%	42.9%
**SD**		0	2.8%	**0**	**0**	**0**
**PD**		11.6%	5.5%	0	9.5%	**0**
**Relapse rate**	-	69.2%	38.9%	84.2%	90.5%	42.9%
**Median PFS 7,5 y**	26%	23%	60%	26%	19%	-
**Median OS 7.5y**	72%	74%	74%	67%	66%	100%

*CR* complete response, *PR* partial response, *SD* stable disease, *PD* progressive disease, *OS* overall survival, *PFS* progression free survival.

### Overall survival and progression-free survival

**Figure 2:**
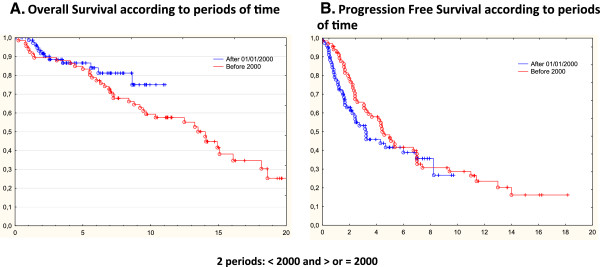
**Disease progression and survival according to the period of time. A.** Overall survival (OS) for two periods of time: <year 2000 and > or equal to 2000. **B.** Progression free survival (PFS) for to two periods of time, < 2000 and > or equal to 2000.

According to the changes in treatment management over time, OS, and PFS were analyzed for two periods of time (associated to the rituximab era): < the year 2000 and > or = 2000. As shown in Figure 2A and 2B, there was no difference in terms of OS or PFS at 5 years for the two time periods (85% vs. 87% and 48% vs. 42% respectively). Finally, we have shown a trend to a better OS at 7.5 years for the patients treated after 2000 (75%) than that for those treated <2000 (59%) but this difference is not statistically significant (*p=*0.29).

**Figure 3:**
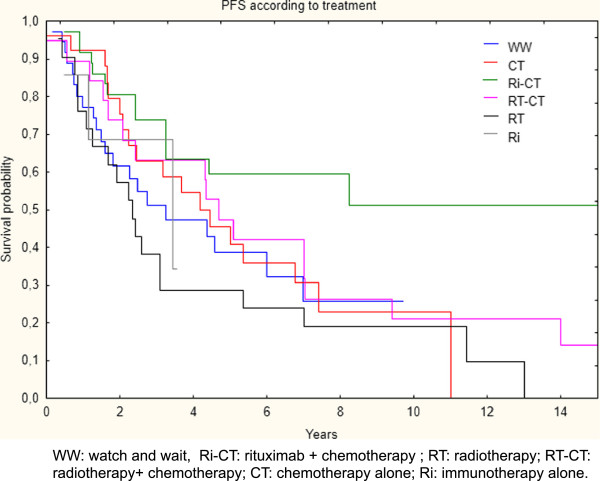
Progression free survival (PFS) according to type of treatment.

When considering PFS and OS according to the type of treatment, analyses did not show any difference in terms of OS at 7.5 years: 72% WW, 74% CT, 74% Ri-CT, 67% RT-CT, 66% RT, and 100% Ri. By contrast, a significant difference was found for PFS at 7.5 years in favor of Ri-CT (60%) versus the others (19% RT, 26% RT-CT, 23% CT, and 26% for the WW strategy; *p=*0.00135), as shown in Figure 3.

Follicular lymphomas are slow-growing tumors with a relapsing and remitting course following treatment and usually result in death from the illness after several years. Although spontaneous regression may occur and progression may be slow, approximately 50% of patients undergo histological transformation to a more aggressive lymphoma with a poor prognosis [13]. In early-stage FL, the prospect of curing or, at least, maintaining long relapse-free survival often leads to treatment initiation [14]. On the other hand, the long life-expectancy of these patients, in spite of the disease, encourages treatment side-effects to be minimized as much as possible [6]. Nevertheless, while approximately 15 to 30% of patients with nodal or extra-nodal FL present clinical Stage I or II disease, therapy for early-stage FL offers a large number of possibilities: watchful waiting, single agent alkylating therapy, radiation therapy, CHOP regimen, and rituximab with various possible combinations. Defining the standard of care remains complex. In this retrospective study, we classified patients into six groups according to the treatments administered: abstention or delayed therapy, chemotherapy alone, radiotherapy alone, a combination of chemotherapy and radiotherapy, immunotherapy, and immunochemotherapy. RT alone is considered as the mainstay treatment showing long survival in some studies [3-5,15]. With a median follow-up period of 8 to 19 years, retrospective studies on RT have shown a 10-year PFS rate of 45% and 10-year OS rate of 65 to 75% [2,3,16,17]. These results do not differ from those observed in this analysis in terms of OS at 10 years (66% for the RT group and 62% for the RT-CT group). On the other hand, in our study, the PFS rate was lower than that reported in literature (around 25%); except for immunochemotherapy (60% PFS at 7.5 years) [3,16,17]. One explanation is the inherent limits of the retrospective setting and that some patients were referred to our center at the time of relapse.

The two issues surrounding radiation are field size and dose. The vast majority of early FL patients treated in retrospective series demonstrated excellent PFS following RT with involved field radiation, with a minority receiving extended field radiation. Recently, a randomized controlled trial of low- versus high-dose RT [18] for indolent non-Hodgkin’s lymphoma demonstrated an equivalence of the two indolent lymphomas including early-stage FL with respect to response rate, PFS, and OS. Additionally, the prospective multicenter MIR (Mabthera and Involved field Radiation) study, which combines anti-CD20 antibody rituximab in combination with involved field radiotherapy (30–40 gray), evaluates the efficacy of rituximab in preventing out-filed recurrences as well as the safety of this combination [19].

Very few studies have looked at CT alone in localized FL [20]. As in our study, PFS did not differ for patients who received CT with or without RT (PFS at 7.5 years 23% for CT vs. 26% for combination RT-CT). The major question is whether immunochemotherapy improves the outcome for patients with early-stage FL. A recent analysis conducted by the National LymphoCare Study revealed, in a large prospectively enrolled group of patients with Stage I FL, that PFS was significantly improved with either rituximab-CT or systemic therapy plus RT when compared to RT alone [21].

## Conclusion

One interesting observation in our study was that delayed therapy was associated with a similar outcome than that observed in treated patients (OS at 7.5 years for the different treatments and WW did not show any difference: 72% for WW compared to 74% for Ri-CT and 67% for RT-CT). This observation was previously reported in two retrospective studies [7,8]; the watchful waiting approach may thus be a reasonable strategy that does not affect OS (85% at 10 years) [22]. These results demonstrate that the choice of first-line treatment has no influence on the course of the disease, in contrast to patients receiving cytotoxic therapy, where the addition of rituximab improves OS [23]. However, when treatment is required, combined immunochemotherapy appears to be the best option. According to Montoto [24] and Friedberg et al. [21], the question of standard treatment in localized FL remains unresolved and requires well-designed randomized clinical trials.

## Patients and methods

### Patient characteristics

We analyzed 145 patients (pts) (79 females and 66 males), with a median age of 55 years (20% of patients aged >65 years) and a diagnosis of early-stage FL made between January 1967 and January 2011. All of these patients are either untreated patients with a diagnosis of FL made in our unit or refered to our unit in the relapse setting. With a median follow-up interval of 7 years and one month, diagnosis was based, in all cases, on a surgical specimen (lymph node or extra-nodal site biopsy). Histological reports provided the diagnosis in accordance with the World Health Organization (WHO) classification system. Patients with a histological WHO classification Grade 3b were excluded, as most experts would recommend a management plan identical to that of diffuse large B-cell lymphoma (DLBCL) [25].

Staging was designated according to the Ann Arbor system with “bulky disease” recording (>7 cm). Initial staging included a thoracic and abdominal CT scan, and a bone marrow aspirate and biopsy. PET CT has only been used in recent years. A complete blood count and routine blood chemistry testing, including LDH and β2-microglobulin levels, were performed. The Follicular Lymphoma International Prognostic Index (FLIPI) score was calculated based on individual parameters [26]. This index is based on five parameters with a poor outcome associated with age ≥60 years, hemoglobin <12 g/dl, LDH above normal range, Stage III/IV disease, and presence of >4 nodal sites, which is subdivided into low (0–1), intermediate (2), and high (≥3) risk groups.

Patients were retrospectively divided into six groups according to the initial therapeutic strategy that was applied: WW, CT alone, RT alone, RT-CT, Ri alone, and Ri-CT. The initial treatment was decided upon by the attending physician following discussion with the patient. Clinical and biological patient characteristics are summarized in Table 1.

### Statistical analysis

Statistical analyses were performed using SPSS software version 12.0. Demographic characteristics were compared across the groups using chi-squared tests for qualitative characteristics, while the *t*-test, Mann–Whitney test, and ANOVA test were used for quantitative characteristics. The event-free survival (EFS) and the OS were calculated according to the Kaplan-Meier product limit estimator method. Survival was calculated from the time of diagnosis to death for any given case. PFS was calculated from the time of diagnosis to the moment of progression or relapse. Univariate comparisons of survival curves were made using the log-rank test*.* Two side *P*-values of <0.05 were considered statistically significant.

## Competing interests

The authors indicated no potential conflicts of interest to declare in relation to this manuscript.

## Authors’ contributions

Conception and design: A-SM, GS, and BC. Provision of study materials or patients: All co-authors. Collection and assembly of data: A-SM, DB, and LL. Data analysis and interpretation: A-SM, LL, GS, and BC. Manuscript writing: A-SM, GS, and BC. Final approval of the manuscript: all co-authors.
